# Implementing Mass Rearing of *Trissolcus japonicus* (Hymenoptera: Scelionidae) on Cold-Stored Host Eggs

**DOI:** 10.3390/insects12090840

**Published:** 2021-09-18

**Authors:** Barbara Bittau, Maria Luisa Dindo, Giovanni Burgio, Giuseppino Sabbatini-Peverieri, Kim Alan Hoelmer, Pio Federico Roversi, Antonio Masetti

**Affiliations:** 1 Department of Agricultural and Food Sciences—DISTAL, University of Bologna, I-40127 Bologna, Italy; barbara.bittau2@unibo.it (B.B.); marialuisa.dindo@unibo.it (M.L.D.); giovanni.burgio@unibo.it (G.B.); 2 CREA, Research Centre for Plant Protection and Certification, I-50125 Florence, Italy; giuseppino.sabbatini@crea.gov.it (G.S.-P.); piofederico.roversi@crea.gov.it (P.F.R.); 3 USDA-ARS—Beneficial Insects Introduction Research Unit, Newark, DE 19713, USA; kim.hoelmer@usda.gov

**Keywords:** exotic species, samurai wasp, biological control, egg masses, *Halyomorpha halys*

## Abstract

**Simple Summary:**

*Halyomorpha halys* (Stål) (Heteroptera: Pentatomidae) is a polyphagous and invasive pest that has been causing severe damage to agricultural systems worldwide including Italy. *Trissolcus japonicus* (Ashmead) (Hymenoptera: Scelionidae), an egg parasitoid native to regions of Asia where *H. halys* originated, seems to be one of the most promising solutions for decreasing pest pressure. In 2020, field releases of *T. japonicus* were approved in Italy with the aim of releasing 120,000 parasitoids. Thus, it was necessary to develop an efficient rearing system to support this biological control program. In this study, some parameters that could influence the rearing of *T*. *japonicus* on cold-stored eggs of *H. halys* were investigated. Refrigeration at 6 °C for as long as several months is an effective method of storage for *H*. *halys* egg masses. Female parasitoids produced the highest number of progeny when exposed to egg masses for 72 h. Adult wasps could also be stored at 16 °C for up to 90 days with a negligible decrease in survival, but a significant decrease in production of progeny.

**Abstract:**

*Halyomorpha halys* (Stål) (Hemiptera: Pentatomidae), a pest of Asian origin, has been causing severe damage to Italian agriculture. The application of classical biological control by the release of *Trissolcus japonicus* (Ashmead) (Hymenoptera: Scelionidae), an exotic egg parasitoid, appears to be one promising solution. In Italy, releases of *T. japonicus* in the field were authorized in 2020. In this study, some parameters that could influence the rearing of *T*. *japonicus* in insectaries were investigated. A significantly higher production of progeny was observed on host eggs stored at 6 °C (86.5%) compared to −24 °C (48.8%) for up to two months prior to exposure to parasitism. There were no significant differences in progeny production from single females in a vial provided with only one egg mass (83.2%) or 10 females inside a cage with 6 egg masses (83.9%). The exposure of parasitoids to refrigerated (6 °C) egg masses of *H*. *halys* for 72 h led to a significantly higher production of progeny (62.1%) compared to shorter exposures for 48 (44.0%) or 24 h (37.1%). A decline in production of progeny by the same female was detected between the first (62.1%) and the second parasitization (41.3%). Adult parasitoids stored at 16 °C for up to 90 days had an 87.1% survival rate, but a significant decrease in progeny production was detected. These parameters could be adjusted when rearing *T. japonicus* for specific aims such as the production of individuals for field release or colony maintenance.

## 1. Introduction

*Halyomorpha halys* (Stål) (Heteroptera: Pentatomidae), also known as brown marmorated stink bug, is an agricultural pest that has become widespread outside its Asian native areas [1,2]. In Italy, severe damage has been recorded since 2015, especially in pear, apple, peach, nectarine, kiwifruit, and hazelnut orchards [3,4].

The initial reaction to *H. halys* invasions has been an increased use of broad-spectrum insecticides, which negatively affect the existing IPM programs [5] and are neither fully effective, nor sustainable [6,7,8]. Therefore, environmentally friendly and self-sustaining methods of managing *H. halys* have been investigated, and the use of natural enemies (biological control) appears a feasible solution [9].

Biological control programs based on the use of natural enemies native to regions of Asia where *H. halys* originated seems to be a promising solution [10]. Surveys for natural enemies of *H*. *halys* in the Beijing region conducted from 2001–2007 [11] found six egg parasitoid species, among which *Trissolcus japonicus* (Ashmead) (Hymenoptera: Scelionidae), was the most abundant. This species was initially reported as a new species, but was subsequently synonymized with *T. japonicus* by Talamas et al. [12]. It is a solitary egg parasitoid of *H*. *halys* and had an average annual parasitism rate of 50–70%, and its sex ratio is strongly female biased [13]. Furthermore, it has been shown that eggs of *H*. *halys* can be stored for long periods at low temperatures, allowing effective mass-rearing of *T. japonicus* under laboratory conditions [14,15,16] which is important for prospective biological control program applications. For these reasons, *T. japonicus* was selected for classic biological programs in Europe and North America [10]

In Italy, while assessments of host-specificity of *T*. *japonicus* were still underway in quarantine laboratories, adventive parasitoid populations were detected in northern regions [17,18]. Based on a risk analysis of *T. japonicus*, a petition for its release in the field in Italy was approved in 2020, with plans to rear and release over 120,000 females in five regions and two provinces in northern Italy [19,20]. Thus, it was necessary to develop an efficient rearing system for insectaries and rearing centers to efficiently support a biological control program to guarantee a high and constant production of parasitoids quickly and ideally at a low cost [21].

Investigations aimed at improving host and parasitoid rearing and their storage are ongoing with the aim of optimizing rearing processes [14,16,22,23]. Focusing on this possibility, the present study aimed at evaluating the following rearing parameters for *T. japonicus*: (a) Storage of *H. halys* egg masses: temperature and length of storage; (b) parasitization patterns: optimal ratio between number of *T. japonicus* females and number of *H. halys* egg masses, length of exposure time of host egg masses to parasitizing *T. japonicus* females, number of optimal oviposition opportunities for individual *T. japonicus* females; and (c) adult parasitoid storage at 16 °C: effects on wasp survival and fertility. All tests were conducted with *H. halys* egg masses under different combinations of storage temperatures and storage times, with the goal of developing a rearing process that guarantees high quality and steady availability of the parasitoid for experiments and for release programs in the field.

## 2. Materials and Methods

### 2.1. Insect Origin and Rearing

The colony of *H*. *halys* was established in 2015 from adults that were field collected in the Emilia-Romagna region. It was maintained in a walk-in climatic chamber (25 ± 2 °C, 50–70% RH and 14 L:10 D photoperiod) at the Department of Agricultural and Food Sciences (DISTAL) of the University of Bologna. Adult stink bugs were kept in Plexiglas cages (50 × 50 × 50 cm) provided on each side with an opening covered with plastic mesh for ventilation. Green beans, carrots, peanuts, soybean seeds, and fresh fruits (pears, apples, and kiwi fruits) were offered as food. A source of water was supplied by wet cotton and paper towels were provided for resting and oviposition sites. Peach seedlings were placed inside the cages for both oviposition and feeding.

Juveniles of all stages were reared in smaller cylindrical plastic boxes (height 30 cm, ø 15 cm), covered with a plastic mesh for ventilation, and fed with green beans, peanuts, and sunflower seeds. Rearing units were checked three times a week to replace food and collect freshly laid egg masses from the adult cages.

Collected egg masses were stored either at −24 ± 1 °C or at 6 ± 1 °C prior to parasitoid rearing bioassays. At either temperature, cold storage prevented eggs from hatching, but they were still suitable for the development of *T*. *japonicus* [22,24,25]. We selected the storage temperature of −24 °C instead of −80 °C as recommended by Wong et al. [22] because ultra-low temperature freezers are not available in most of our insectaries. Storage at 6 °C was chosen because it is well below the lowest developmental threshold of *H*. *halys* (12–13 °C) [24], thus it disrupts stink bug embryogenesis without the need for freezing [22]. Before storage, egg masses were carefully inspected under a stereomicroscope and only those appearing viable were used for bioassays and parasitoid rearing. Egg masses that showed detrimental abnormalities (e.g., incomplete number of eggs per egg mass, eggs laid haphazardly on the walls of the cages, egg masses containing partially cannibalized eggs) were entirely discarded.

The colony of *T. japonicus* was started in May 2020 with adult wasps emerging from parasitized eggs of *H*. *halys* provided by CREA—Plant Protection and Certification in Florence (authorization no. DG/DISR/DISR05/0013647-19 April 2018) and transferred under authorization DG/DISR/DISR05/0013952-29 April 2020. The investigated strain of *T. japonicus* originated from a “Beijing” colony of the USDA-ARS Beneficial Insects Introductions Research Unit, in Newark, DE, USA that has been maintained since its collection in Beijing (China) in 2007.

For routine parasitoid rearing, each female wasp was isolated in a plastic vial (height 10 cm, ø 2 cm) closed with a mesh cap. The wasps were fed daily with honey drops applied to the cap mesh and provided with a single egg mass of *H*. *halys* for 72 h at 25 ± 1 °C, 50–70% RH, and 16 L:8 D photoperiod. These conditions allowed a new parasitoid generation to be produced in 10–11 days [11]. A standard exposure time of egg masses (that had been stored at 6 °C prior to parasitism) to female wasps for 72 h was chosen to ensure the maximum possible parasitization. After exposure, egg masses were moved to clean vials and held for the development of parasitoids at the conditions described above. The newly emerged parasitoids were kept together for 10 days to allow mating. Mated female parasitoids produce female biased progeny (≈90%/female); generally, one or two males emerge from each egg mass [13,14]. Only two egg masses were offered to each female during its lifetime (life expectancy: 25–30 days at 25 °C or 55–60 days at 21 °C; fed with honey) because additional exposures would not be worthwhile considering the decline in offspring production over time [14,26].

### 2.2. Standard Protocol Used across All Experiments

Parasitoids from the same egg mass were maintained together for 10 days after emergence in a single vial at 25 ± 1 °C, 50–70% RH, and 16 L:8 D photoperiod. The occurrence of at least one male was always ensured, thus all females used in the experiments were presumably mated. Adult parasitoids were fed only with honey as previously described.

Before being supplied to wasps, the egg masses were examined and discarded if any collapsed or blackened eggs were found. Exposure of egg masses for parasitization was carried out in a climatic chamber set at 25 ± 1 °C, 50–70% RH, and 16 L:8 D photoperiod. Parasitized egg masses were held at the same conditions until the emergence of parasitoids. The progeny production was calculated as the percentage of emerged adult parasitoids out of the total number of eggs exposed to parental females.

### 2.3. Effect of Storage of H. halys Egg Masses on Parasitization by T. japonicus

Two experiments were carried out to investigate the effects of storage temperature and duration of storage of *H*. *halys* egg masses prior to their parasitization on the subsequent production of parasitoid progeny.

#### 2.3.1. Effects of Pre-Exposure Storage Temperature of the *H. halys* Egg Masses on Parasitoid Progeny Production

Frozen and refrigerated egg masses of *H*. *halys* were compared. After collection, the egg masses were stored at −24 ± 1 °C for 76 days (*n* = 98) between May and July 2020 or at 6 ± 1 °C for 60 days (*n* = 92) between March, May, and July, 2020. Before exposure to wasps, egg masses were individually glued with vinylic adhesive to a piece of paper and kept at room temperature for one hour. For parasitization, each *T*. *japonicus* female was isolated in a vial and provided with a single egg mass for 72 h.

#### 2.3.2. Duration of Storage of *H. halys* Egg Masses at 6 °C

For this experiment, 92 freshly collected egg masses were stored at 6 ± 1 °C, half of them for 50 days, the others for 28 days. Two egg masses, one stored for 50 days and the other stored for 28 days, were provided to the same female *T*. *japonicus* in a test vial at the same time. Exposure lasted for 72 h.

### 2.4. Exposure of Egg Masses to Parasitoids

Three experiments were performed to evaluate different procedures for egg mass exposure to *T*. *japonicus* female(s) for parasitization.

#### 2.4.1. Individual vs. Group Parasitization

For this test, females held individually in plastic vials were compared with groups of 10 females of the same age housed in nylon cages (30 × 30 × 30 cm). The wasps were fed with honey drops applied to the mesh of the vial cap and as droplets on a piece of waxed paper in the cages. All egg masses used in this experiment were stored at −24 ± 1 °C for 98 days before exposure. A single egg mass was provided to each female wasp in the vials, while 6 egg masses in a Petri dish lid were placed with females in each cage. The exposure was carried out for 72 h in both cases. The experiments were performed three times during July and August 2020. In each replicate, 24 vials (24 parasitoids with 24 egg masses) and four cages (40 parasitoids with 24 egg masses) were used.

#### 2.4.2. Duration of Exposure of Egg Masses to *T. japonicus* Females

Three different exposure durations to females isolated in vials were compared: 24, 48, and 72 h. An egg mass was supplied to each female for one of the three exposure durations. Egg masses (*n* = 118) were stored at 6 ± 1 °C for 28 to 50 days before use. Sixty egg masses were exposed for 24 h, 29 for 48 h, and 29 for 72 h.

#### 2.4.3. First vs. Second Oviposition by *T. japonicus* Females

The number of progeny produced by an individual female wasp during its first and second oviposition exposures were compared. For the first exposure, naive females were isolated in plastic vials and supplied with an egg mass (stored at 6 ± 1 °C for 60 days prior to parasitization) for 72 h. Four days after the first oviposition exposure, a second egg mass (stored at 6 ± 1 °C for 60 days prior to parasitization) was exposed to each parasitoid female for 72 h.

### 2.5. Effect of Length of Storage of Adult Parasitoids at 16 °C on Their Survival and Production of Progeny

We tested the effects of the length of storage on survival and parasitization of *T*. *japonicus* females stored for different periods at 16 ± 1 °C, 50–70% RH, and 16 L:8 D photoperiod. Storage of parasitoids at low temperatures can be an effective method to extend their life and to provide a consistent supply for biological control programs [27]. Storage at 16 °C was selected for our study based on the findings of Cira et al. [23].

#### 2.5.1. Effects on Survival

Before storage, all adult parasitoids used in this experiment were maintained in vials at 25 ± 1 °C, 50–70% RH, and 16 L:8 D photoperiod for 10 days after emergence. In a sample of 25 vials (637 parasitoid individuals in total) survival was recorded without any prior storage at 16 °C (hereafter referred to as 0 days of storage). This was compared with survival in vials stored for either 50 days (*n* = 50, 1352 total parasitoids) or 90 days (*n* = 25, 610 total parasitoids) at 16 °C. During storage, all parasitoids that emerged from the same egg mass were kept together in the same vials and provisioned weekly with honey drops applied to the mesh on the vial caps. Vials were maintained at room temperature for 1 h before mortality assessment.

#### 2.5.2. Effects on Progeny Production

The production of progeny was compared for non-stored females and females stored for 50 days and 90 days at 16 °C. For each period, 30 females were randomly selected from the vials used in the previous experiment. Each of these females was isolated in a vial and supplied with an egg mass that had been previously stored at −24 ± 1 °C for approximately nine months. Indeed, no other eggs were available for these assays, because the egg masses stored for shorter periods either at 6 or −24 °C were used to produce parasitoids to be released for the biological control program.

### 2.6. Data Analysis

Generalized linear models (GLM) with binomial error distribution and probit link function were used to analyze the production of progeny by parasitoids and adult wasp survival. When a factor with more than three levels was analyzed, multiple comparisons among levels were carried out with sequential Bonferroni adjustment.

A generalized linear mixed model (GLMM) with binomial error distribution and probit link function was carried out to analyze the effect of storage time of egg masses at 6 °C on the production of progeny. Females were included as subjects and the two periods of egg storage were considered as within-subject factors. A diagonal correlation matrix was selected. Statistical analyses were performed using IBM SPSS Statistics (ver. 26).

## 3. Results

### 3.1. Effect of Storage of H. halys Egg Masses on Parasitization by T. japonicus

#### 3.1.1. Effects of Pre-Exposure Storage Temperature of the *H. halys* Egg Masses on Parasitoid Progeny Production

**Figure 1:**
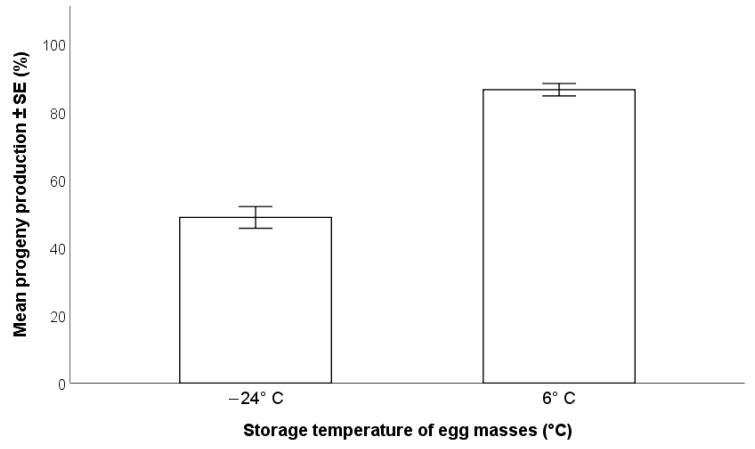
Effects of pre-parasitization storage temperature on the production of progeny by *Trissolcus japonicus* (−24 °C = 76 days; +6 °C = 60 days) (GLM, *p* < 0.001).

*Trissolcus japonicus* females accepted egg masses stored at −24 °C for 76 days and at 6 °C for 60 days, and parasitoid progeny were able to complete development in eggs that had been stored at both temperatures. A higher percentage of adult parasitoids (Wald χ^2^_(1, n=190)_ = 90.93; *p* < 0.001) emerged from eggs stored at 6 °C (86.5 ± 1.8%) than from eggs held at −24 °C (48.8 ± 3.2%) (Figure 1).

#### 3.1.2. Duration of Storage of *H. halys* Egg Masses Storage at 6 °C

**Figure 2:**
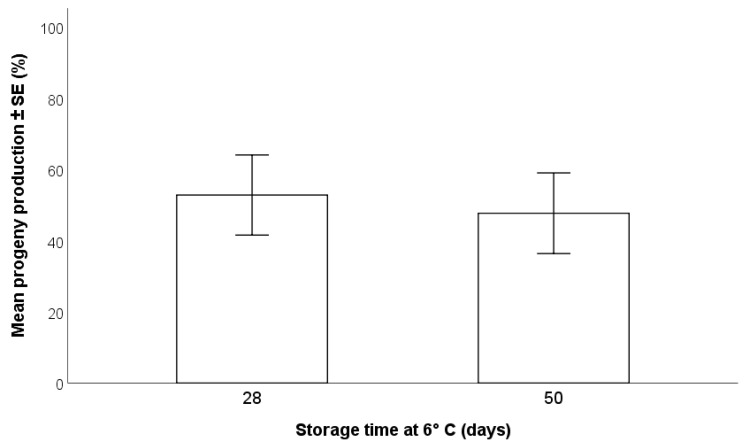
Effects of storage time at 6 °C on the production of progeny by *Trissolcus japonicus* (GLMM, *p* > 0.05).

*Trissolcus japonicus* females oviposited on egg masses that had been stored for either 28 or 50 days, and parasitoids were able to complete their development in eggs stored for both periods. The GLMM did not show any significant effect of storage time of *H*. *halys* egg masses at 6 °C on the progeny emerged (F_(1,28)_ = 0.06; *p* = 0.81). The percent progeny production from egg masses stored for 28 days (52.8 ± 11.3%) was slightly higher than from those stored for 50 days (47.7 ± 11.3%) (Figure 2).

### 3.2. Exposure of Egg Masses to Parasitoids

#### 3.2.1. Individual vs. Group Parasitization

**Figure 3:**
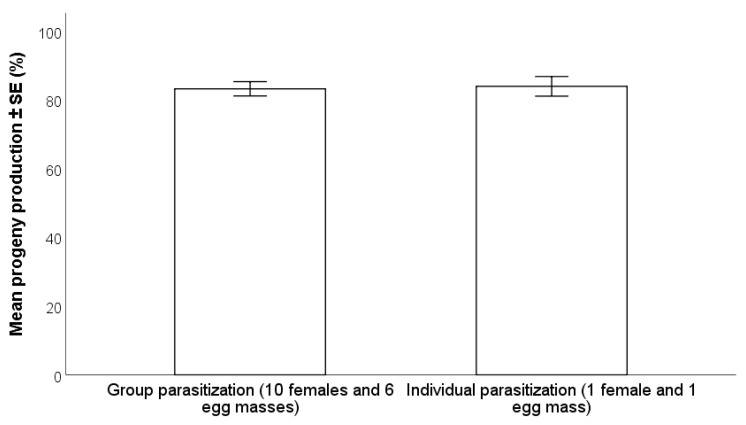
Effect of number of female: egg mass (−24 °C for 98 days) ratio on the production of progeny by *Trissolcus japonicus* (GLM, (*p* > 0.05).

The female:egg mass ratio did not have a significant effect on progeny production (Wald χ^2^ _(1,n=145)_ = 0.16; *p* = 0.90). The percentage of adult parasitoids (83.2 ± 2.1%) that emerged in vials with one female to one egg mass ratio was very similar to that from cages with 10 females and six egg masses (83.9 ± 2.9%) (Figure 3).

#### 3.2.2. Duration of Exposure of Egg Masses to *T. japonicus* Females

**Figure 4:**
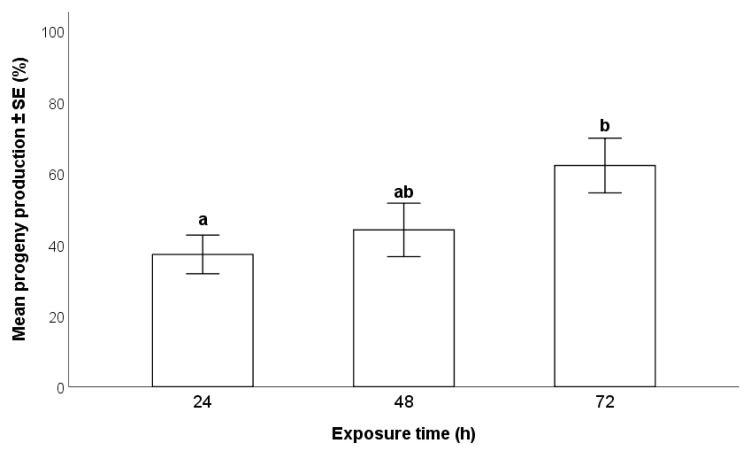
Effects of exposure time of an egg mass (stored prior to exposure at 6 °C for 28–50 days) to a single *Trissolcus japonicus* female on the production of progeny (GLM, *p* < 0.05). Different letters indicate significant differences (*p* < 0.05) as detected by multiple comparison with sequential Bonferroni adjustment.

The length of exposure of an egg mass to *T. japonicus* had a significant effect on the production of progeny (Wald χ^2^ _(2,n=118)_ = 6.77; *p* = 0.04). There was a significant difference between 72 and 24 h exposures, with the percentage of *H. halys* eggs producing parasitoids of 62.1 ± 7.7% and 37.1 ± 5.4%, respectively. Progeny production from 48 h exposures (44.0 ± 7.5%) was intermediate between 24 h and 72 h exposures (Figure 4).

#### 3.2.3. First vs. Second Oviposition by *T. japonicus* Females

**Figure 5:**
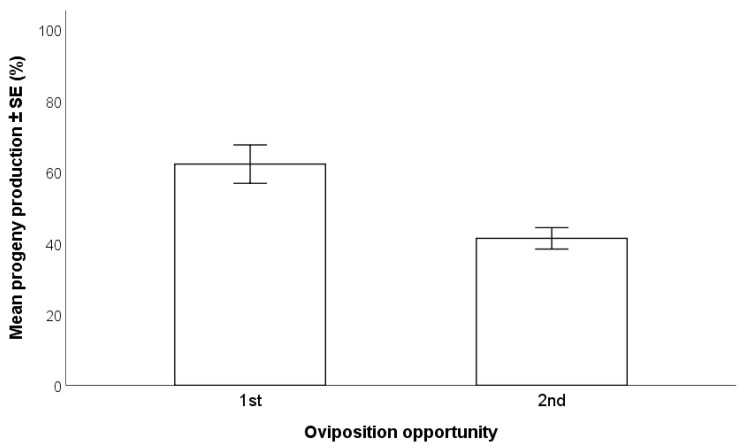
Effects of oviposition opportunity for the same *Trissolcus japonicus* females on the production of progeny (GLM, *p* < 0.001).

A significantly higher percentage of adult parasitoids (Wald χ^2^_(1, n = 212)_ = 14.27; *p* < 0.001) emerged from egg masses parasitized by females during their first oviposition opportunity (62.1 ± 5.4%) than during their second oviposition at four days later (41.3 ± 3.0%) (Figure 5).

### 3.3. Effect of Length of Storage of Adult Parasitoids at 16 °C on Their Survival and Production of Progeny

#### 3.3.1. Effects on Parasitoid Survival

**Figure 6:**
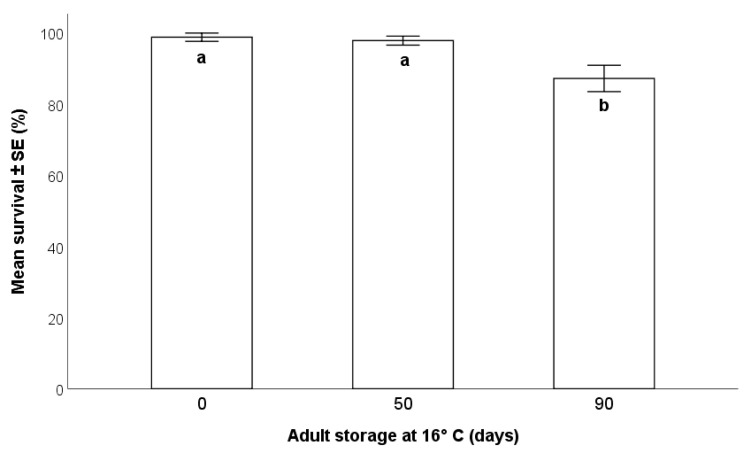
Effects of the length of storage at 16 °C on *Trissolcus japonicus* survival (GLM, *p* < 0.001) Different letters indicate significant differences (*p* < 0.05) as detected by multiple comparison with sequential Bonferroni adjustment.

The survival of *T. japonicus* adult females decreased significantly as the duration of storage increased (Wald χ^2^_(2,n=100)_ = 54.98; *p* < 0.001). A statistically significant difference was detected between 90-day storage (87.1 ± 1.1%) and the other two periods (0 days: 98.7± 0.6%; and 50 days 97.7 ± 0.6%). A difference between 0 and 50 days was not supported by sequential Bonferroni multiple comparison (*p* = 0.37) (Figure 6).

#### 3.3.2. Effects on Progeny Production

**Figure 7:**
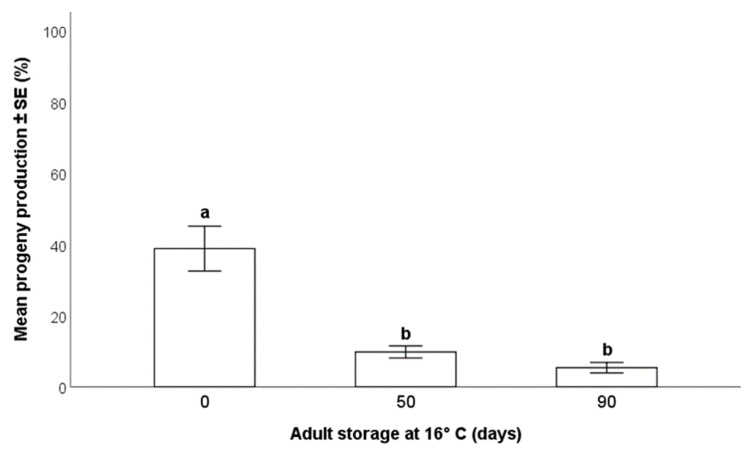
Effects of the length of storage at 16 °C of *Trissolcus japonicus* adults on the production of progeny (GLM, *p* < 0.001). Egg masses of *Halyomorpha halys* were stored at −24 °C for ≈9 months prior to exposure to wasps. Different letters indicate significant differences (*p* < 0.05) as detected by multiple comparison with sequential Bonferroni adjustment.

*Trissolcus japonicus* females stored at 16 °C for up to 90 days retained some capability to parasitize *H*. *halys* eggs, but longer storage periods caused a significant decrease in the production of progeny (Wald χ^2^_(2,N=120)_ = 39.40; *p* < 0.001) (Figure 7). A higher percentage of adult parasitoids (38.8 ± 6.3%) emerged from eggs parasitized by females stored at 16 °C for 0 days than from eggs parasitized by females stored either for 50 (9.8 ± 1.1%) or 90 days (5.4 ± 1.5%). The difference in the emergence rates between 50 and 90 days of storage was not statistically significant (sequential Bonferroni *p* = 0.16).

## 4. Discussion

For successful biological control programs, the optimization of rearing procedures for beneficial organisms is crucial. The storage methods for the requisite host stages and the biological control agent are also paramount [21]. In this study, we focused on some parameters that could influence the progeny production of *T*. *japonicus* reared on cold-stored eggs of *H. halys*.

Stockpiling eggs by storing them at 6 °C was tested as a method to stop the embryogenesis of *H. halys* and was reported to be as effective as freezing them, but with fewer negative effects on successful emergence of parasitoid offspring [16,22]. Our findings confirmed that a period of refrigeration at 6 °C ensured both the death of stink bug embryos and a high level of production of progeny. Egg masses stored at −24 °C were also suitable for *T*. *japonicus*, but lower progeny productions were found in comparison with storage at 6 °C. This is in line with several studies that pointed out the negative effects of freezing egg masses on the quantity and quality of *T*. *japonicus* progeny [16,22]. Freezing may reduce the acceptance of host and egg laying by female parasitoids [16]. Lethal and sub-lethal effects on parasitoid eggs or larvae inside frozen stink bug eggs could also have contributed to the decrease in the emergence of *T*. *japonicus* [16,28]. Ludwick et al. [29] found that egg masses stored at −20 °C produced significantly fewer adults of *T*. *japonicus* in comparison with egg masses stored at −80 °C. On the other hand, Wong et al. [22] reported that progeny productions were higher in eggs refrigerated at 8 °C than in those frozen at −80 °C. Simultaneously comparing refrigeration, freezing, and ultra-low temperature freezing of egg masses in a single study would be useful to identify the most suitable storage conditions as well as identify the best alternatives for laboratories that lack equipment for ultra-low temperature storage.

Storage of up to two months at 6 °C did not negatively affect the suitability of egg masses for parasitization. However, the production of progeny from eggs stored at 6 °C was overall much lower (50.2 ± 7.9%, pooling both 1- and 2-months storage) than what was found in the first experiment comparing 6 °C vs. −24° C (86.5 ± 1.8%) with similar time of storage. This might be due to the different experimental conditions for these experiments (i.e., the provision of two egg masses to a single *T*. *japonicus* female for 72 h (experiment 1.2) and only one egg mass for 72 h (experiment 1.1)). Individual female parasitoids likely divided their ovariole egg load between the two egg masses irrespective of their prior storage periods.

From our results, it appears that the best storage temperature of *H*. *halys* eggs over the short and medium term (up to two months) is refrigeration at approximately 6 °C. However, we did not test storage periods exceeding two months, and it has been shown that egg quality is considerably reduced beyond this time [22].

Increased levels of progeny production can also be obtained by manipulating the parasitoid female:host egg mass ratio during the parasitization process by *T*. *japonicus*. In the third experiment, we evaluated whether parasitization was more effective when one egg mass was exposed to a single female, or when more egg masses were exposed at the same time to several females together in the same cage. We surmised that if an isolated female was infertile or otherwise incompetent even if mated, the entire *H. halys* egg mass would be wasted, whereas, in group parasitization, a failure by one or more females could be compensated by the others. However, our results showed no significant differences between the two methods. A possible explanation of the lack of significant differences could be the guarding behavior of a dominant female that parasitizes an egg mass in the cage. This female could remain on the mass and display aggressive actions toward other parasitoids [30]. Another possible explanation is that virtually all parasitoid females tested individually were able to parasitize. Irrespective of the reasons, the lack of a significant difference means that the choice between solitary and group parasitization can be based on considerations of time, space, and other resources available to a specific rearing facility.

The sex ratio of the progeny was not considered in the present work, however, it may be appropriate to evaluate it in a future study, as there might be significant differences between individual and group parasitization. If multiple females parasitize the same egg mass, and each female lays one or two haploid eggs, it is more likely that a greater number of males will be produced than by solitary female parasitization. However, the guarding behavior of *T*. *japonicus* females might also offset any detrimental effect on sex ratio due to multiple oviposition by different individuals.

A 72-h exposure appeared to be the best option, as a significantly higher production of progeny was obtained when egg masses were exposed for 72 h compared to those exposed for 48 and 24 h. The use of egg masses stored at 6 °C for 1–2 months may have influenced the results of this experiment. Previous studies showed that egg refrigeration guarantees their conservation and successful parasitization by *T. japonicus*, but the overall results were lower when compared to the use of fresh eggs (<3 days old), which are considered optimal [13,14,16].

The exploitation of reproductive females for parasitization of multiple egg masses can be advantageous for producing additional progeny without waiting for the subsequent generation. However, in this study, we found a significant reduction between the first and second oviposition exposures by the same female. This is in accordance with other studies that reported a progressive decline in oviposition over time [13,14]. Sabbatini-Peverieri et al. [14] showed that females deprived of host eggs for seven days accumulated a new load of eggs in their ovaries, thus obtaining high parasitization rates in a subsequent oviposition cycle. This suggests that *T*. *japonicus* is synovigenic [14]. In our experiment, a second egg mass was offered to the females just four days after the first one. It is therefore possible that the discrepancy is due to the short time available to the female to accumulate additional eggs. A mass-rearing system aimed at producing large numbers as quickly as possible for release would thus best exploit females only for a single exposure for parasitization or carry out repeated parasitizations separated by at least one week from each other.

In a rearing system, the scalability of insect production is paramount. With cold storage of adult parasitoids, it is possible to provide an adequate supply of viable insects ready to be released at the right moment [23,27]. A high survival rate was detected for storage periods up to 90 days at 16 °C. Therefore, our findings confirmed that cold storage at 16 °C can be used to extend the lifespan of *T*. *japonicus* [13,23].

However, unlike survival, a notable decrease in progeny production was found after 50 and 90 days of storage. These results differ considerably from those of Cira et al. [23], who did not find any significant differences in parasitoid emergence between *T*. *japonicus* females stored at 23 °C, 18 °C, and 13 °C. However, these authors utilized different photoperiods during the periods of cold storage (10:14 at 13 °C and 18 °C; 16:8 at 23 °C), potentially inducing a dormancy effect in the adults.

They also used *H. halys* egg masses frozen at −80 °C and no older than 109 days, which could have affected the emergence of new adults. Cold storage of *T*. *japonicus* females under our experimental conditions could also ensure the continuity of the rearing over the long-term, but individuals undergoing this treatment are less optimal for field release as they produce fewer progeny than recently emerged parasitoid females.

It is noteworthy that egg masses provided to parasitoids in our final experiment were stored for approximately nine months at −24 °C. When these egg masses were provided to newly emerged parasitoids, comparable productions of progeny were found in eggs stored at −24 °C for 76 days (48.8 ± 3.2%) and in eggs stored for nine months (38.8 ± 6.3%). Although a statistically valid comparison could not be carried out because the two experiments were performed at different times, we hypothesize that the most significant decline in suitability of *H. halys* eggs to *T. japonicus* occurred because of freezing. However, after this initial decrease, egg masses stored at −24 °C seemed to remain suitable for parasitization for several months. This could be exploited to collect and store egg masses over an extended period of time and then use them during periods with high requirement for adult parasitoids.

## 5. Conclusions

From our findings, we can derive a few rearing recommendations:

(i) In terms of parasitoid production, the most effective method of storage for *H*. *halys* egg masses is refrigeration at 6 °C for a maximum of two months; beyond this time, a progressive decline in egg suitability has been shown [22]. Freezing may be considered for long term maintenance of *H*. *halys* egg masses, but negative impacts on *T*. *japonicus* production of progeny were detected.
(ii) A 72-h exposure time of *H*. *halys* egg masses to parasitoids resulted in higher progeny production compared to 24 and 48 h, especially with wasps at their first oviposition opportunity. There were no differences between individual and group parasitization.
(iii) The storage of the adult parasitoids at 16 °C allowed for their survival up to 90 days from emergence, but this was accompanied by a progressive decrease in the production of progeny. Consequently, the long-term conservation of adults at low temperatures may be suitable for maintenance of the rearing during less-demanding periods, but not for maximal production of parasitoids to be released in biological control programs.

## Data Availability

Not applicable.
